# Testosterone causes pleiotropic effects on cleanerfish behaviour

**DOI:** 10.1038/s41598-019-51960-w

**Published:** 2019-11-01

**Authors:** Marta C. Soares, Renata Mazzei, Sónia C. Cardoso, Cândida Ramos, Redouan Bshary

**Affiliations:** 1 CIBIO, Centro de Investigação em Biodiversidade e Recursos Genéticos, Universidade do Porto, Campus Agrário de Vairão, 4485-661 Vairão, Portugal; 2 Université de Neuchâtel, Institut de Biologie, Eco-Ethologie, Rue Emilie-Argand 11, 2000 Neuchâtel, Switzerland; 3 Museu Virtual da Biodiversidade, Universidade de Évora, Casa Cordovil, 2º Andar, rua Joaquim Henrique da Fonseca, 7000-890 Évora, Portugal

**Keywords:** Behavioural ecology, Physiology

## Abstract

Mathematical modelling regarding evolutionary theory typically assumes that optimal strategies are not constrained through mechanistic processes. In contrast, recent studies on brain anatomy and neurobiology suggest that flexibility in social behaviour is rather constrained by the physiological state of the social decision-making network. Changing its state may yield selective advantages in some social contexts but neutral or even detrimental effects in others. Here we provide field evidence for such physiological trade-offs. We subjected wild female cleaner wrasse to injections of testosterone or of saline solution (control) and then observed both intraspecific interactions and interspecific cleaning behaviour with other reef fish, referred to as clients. Testosterone-treated females intensified intraspecific social interactions, showing more aggression towards smaller females and tendencies of increased aggressive and affiliative contacts with dominant males. Such testosterone-mediated changes fit the hypothesis that an increase in testosterone mediates female’s focus on status in this protogynous hermaphrodite species, where females eventually change sex to become males. Moreover, we also identified other effects on interspecific social interactions: testosterone-treated females interacted less with client reef fishes and hence obtained less food. Most importantly, they selectively reduced service quality for species that were less likely to punish after being cheated. Overall, our findings suggest that testosterone causes pleiotropic effects on intra and interspecific social behaviour by broadly influencing female cleaners’ decision-making.

## Introduction

Research on the diversity, evolution and stability of cooperative behaviour has been successful in generating solutions as to why individuals cooperate by investing (behaviour that reduces the immediate payoff of the actor) in the provisioning of benefits to other individuals. Indeed, a large variety of game theoretical concepts explain the evolution and stability of cooperation^1,2^. However, these functional theoretical approaches have so far mostly explored rather artificial and simplistic strategies that do not seem to correspond to how animals make social decisions. Models rarely incorporate learning mechanisms^3^ or consider emotions that modify decisions, for example due to pair bonding (‘friendships’^4^). Instead, when it comes to cooperation, models traditionally focus on how cooperation may prevail in the presence of defectors but do not impose trade-offs or constraints on behaviour, as is typically assumed in other fields of behavioural ecology^5^. It is therefore an important next step to integrate mechanisms into the functional approach in order to understand how brain functioning and physiology may help or hinder the expression of cooperative behaviour within and between species^6,7^.

A key recent neurobiological insight suggests that all vertebrates share a so-called social decision-making network which consists of various brain areas identified as being crucially involved in the modulation social behaviour (aggression, affiliation, bonding, parental behaviour, social stress), sharing connections between nuclei^8,9^. Androgens and other steroid receptors are present in different nuclei of this network, suggesting that overall state of the network and therefore animal social decisions are modulated by these hormones^10^. The best studied androgen in both male and female vertebrate species is testosterone^11^, which has a multivariate regulatory role in behaviour. Indeed, accumulated evidence has demonstrated that testosterone has the isolated capacity to underlie changes to individuals’ behaviour, but social and physical environment are also crucial elements, thus allowing for the development of reciprocal interactions between androgens and behaviour [i.e., biosocial model^12^; challenge hypothesis^13^]. Hence, context may impact the functional consequences of changes in testosterone, whether transient or long term, requiring mechanisms that can translate and integrate multi-modal social information relevant to the organism, with these translating into neuroendocrine activity responsible for the production of testosterone^14^. Specifically, these induced changes in testosterone will enhance variations in the signalling cascade of this androgen potentially by either: (a) acting on steroid binding proteins that mediate the availability or density of the hormone to receptors at the target tissue, (b) via conversion to other biologically active steroids by specific enzymes or (c) by transcription of co-activators and co-repressors, changing the genomic action of steroids^15,16^. Thus, receptor activation by androgens can have rapid stimulation within seconds to minutes (rapid response by neurosteroids at the brain level) to genomic or more structural effects happening in wider temporal intervals^16^. Testosterone mechanisms are also notably acknowledged to affect competition for resources and social status^17,18^. Its application in economic/cooperative-related contextual tasks revealed apparently conflicting results. In some studies, testosterone seems to increase punishment of defectors, in-group cooperation and fairness^19–22^, while in others it seems to lower generosity and trust^23,24^. These discrepancies may be attributed to variation in methods (i.e. correlations with endogenous testosterone levels versus administrated exogenous testosterone) and also to subject sex (subjects could be female, male, or both). But more generally, it emerges from various studies in which testosterone actions are often related to own perception of status, that under some circumstances increased level of testosterone may lead to increased competitive behaviour, while other situations lead to increased social concern and cooperative behaviour^13,18,20,25^.

The arising question is whether testosterone acting on the social decision-making network mediates coherent adjustments in social behaviour across contexts (like alternative life history strategies leading to a suit of adaptive differences) or instead, with selective effects in one social context (for instance, increased aggressiveness against a competitor) that cascade into another social context (such as, reduced cooperativeness with a partner). The former scenario would fit an adaptationist theoretical approach that may discard constraints while the latter scenario emphasises the need to study the evolution of mechanisms as a higher order of organisation for decision-making^26^. For testing these hypotheses, we require experimental (exogeneous) manipulation of testosterone and to measure its effects on behaviour in different social contexts, ideally with opposing goals. The acute increase of circulating testosterone levels is aimed to induce rapid changes in behaviour by acting on neural pathways that involve the engagement of steroids with membrane receptors and/or with intracellular signaling circuits^27,28^. A suitable study system is the marine cleaning mutualism involving the Indo-Pacific bluestreack cleaner wrasse *Labroides dimidiatus*. The cleaners have been mostly studied due to their interspecific interactions with so-called ‘client’ reef fishes, which visit cleaners to have ectoparasites removed^29^. Conflict arises because cleaners prefer to eat client mucus, which constitutes cheating^30^. Cheating acts correlate with observable jolts performed by clients in response to cleaner mouth contact^31^. This conflict has led to the establishing of various sophisticated social strategies in both clients and cleaners. Clients may either punish cleaners, switching to a different one in response to being cheated or simply to avoid visiting these biting cleaners by observing them beforehand, e.g. observation of ongoing interactions between cleaners and third parties^32–34^. Cleaners, on the other hand, adjust their service quality to the strategic options of each client species, client size, and the presence of bystanders that could observe current interactions^33,35^. Service quality, i.e. levels of cooperation, is not only a function of cheating frequency but also of interaction duration and the amount of simple physical contact, known as tactile stimulation, that a cleaner provides with its pelvic fins^36^. Tactile stimulation lowers cortisol levels in clients^37^. Here, we focused on how testosterone might affect cleaners’ decisions, in relation to their interspecific social context (e.g. cleaning service quality). However, we expect that primary effects of testosterone should also occur at the intraspecific social behaviour level^11^.

*Labroides dimidiatus* individuals also have an interesting social life: they are protogynous hermaphrodites, starting their reproductive life as females and eventually changing sex to become male and obtaining a harem of females^38^. Because female fish are, in some circumstances, able to express higher levels of testosterone compared to those of males^39,40^, the interpretation of its role in fish may differ from other vertebrate groups. A possible role for females (like those of males) lies in the indeterminate growth of fish throughout their life span, which is dependent on testosterone levels as one main factor^41^. This applies to female *L*. *dimidiatus* cleaner wrasses which are known to increase in social dominance in accordance to body size^42^, because it is usually the largest (and more dominant) female that changes sex and take over the harem if or when male disappear^43^. In addition, males commonly live and clean in pairs, choosing the largest female of his harem^42^, with those pairs getting access to larger clients as these provide better cleaning service to clients^44^. Thus, for females, it should pay to increase in dominance and social status and eventually to jointly occupy the same cleaning station with the male, as to gain access to better foraging opportunities even if sometimes they need to agonistically interact with other females^45^. In this case, rises in female testosterone levels would be expected to accompany female social status increase.

However, a putative increase in testosterone levels and as a consequence in body size, could become a limiting factor for females. For instance, male punishment of female cheating behaviour during client inspection becomes more severe when male and female cleaners are closer in size (e.g. male chases increase towards larger and thus more socially dominant females)^45^. Considering that testosterone has an important role in dominance-related behaviours, here we aimed to discover how the putative experimental induction of a better statutory position via testosterone administration would affect wild female cleaner wrasses’ strategic interactions with male partners and with other females. Following the existing literature, we expected that testosterone-induced increase of perceived dominance generates a rise of strategic competition towards other conspecifics. Furthermore, we asked whether such competitive status-seeking would interfere with the female’ behaviour when interacting with client species. If status-seeking introduces time budget trade-offs, we expected that testosterone leads to a decrease in time spent interacting with clients. Most importantly, we asked whether testosterone would affect cleaners’ cooperative levels. We expected that inspection quality would either be unaffected or that cleaners would switch to a short-term payoff maximizing strategy, which according to various evidence consists of being rather cooperative with small clients and exploiting larger clients^34,46^.

## Results

### Effects of testosterone on conspecific-directed behaviour

**Figure 1:**
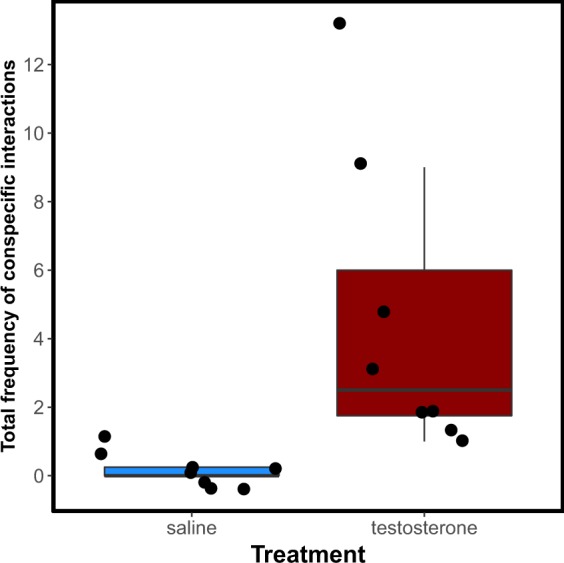
Total frequency of conspecific interactions for cleaner fish treated with saline (blue box) and testosterone (red box).

**Figure 2:**
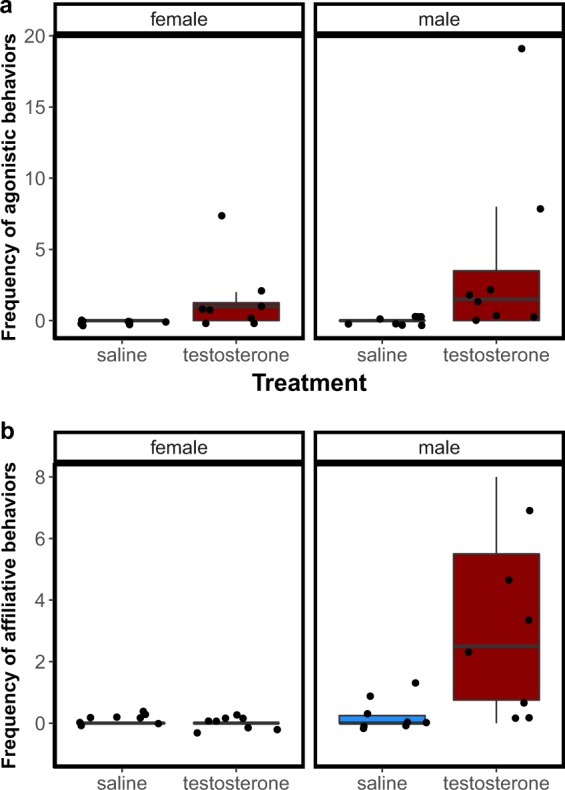
Frequency of (**a**) agonistic and (**b**) affiliative behaviors displayed by cleaner fish treated with saline (blue boxes) and testosterone (red boxes) towards female and male conspecifics. Agonistic behaviors = sum of chases and attacks to a conspecific; Affiliative behaviors = sum of interspecific cleaning interactions, poses to a conspecific and swims with a conspecific.

All manipulations took place in the wild (Lizard Island, Australia) so that all interactions could arise between real fish. A total of 16 female cleaner fish were tested: a single dose of 2 µg of testosterone or saline solution (control) was administrated exogenously (intramuscularly) on each individual’ territory (handling never took more than 3 minutes) and then videotaped for the next 45 minutes. In general, cleaners treated with testosterone interacted significantly more with conspecifics than control cleaners did (W = 2, p-value = 0.0013) (Fig. 1). Treated cleaners displayed significantly more agonistic behaviors than control cleaners towards other females (Agonistic behaviors = sum of chases and attacks to a conspecific; W = 12, p-value = 0.012, corrected p-value = 0.037, Fig. 2a; see methods for further information). Subjects never showed affiliative behaviours towards other females, independently of treatment (Affiliative behaviors = sum of interspecific cleaning interactions, poses to a conspecific and swims with a conspecific; Fig. 2b; see methods for further information). Towards males, treated females tended to show both increased agonistic behaviors (W = 16, p-value = 0.032, corrected p-value = 0.063) (Fig. 2a) and an increased frequency of affiliative behaviors (W = 14, p-value = 0.043, corrected p-value = 0.063) (Fig. 2b).

### Effects of testosterone to the quality of service provided to clients

**Figure 3:**
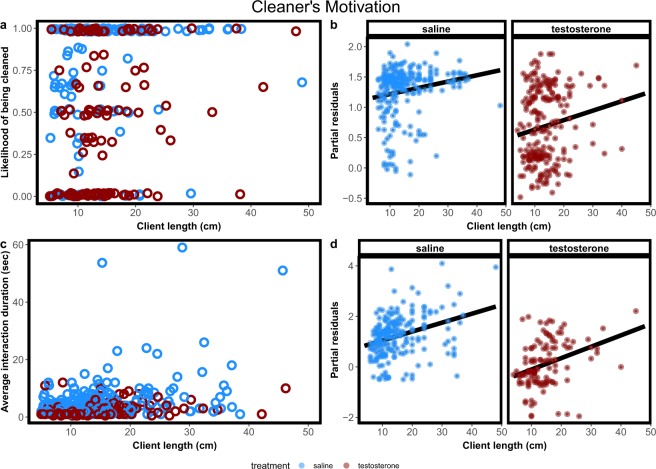
Cleaners motivation measured as (**a**,**b**) client likelihood of being cleaned and (**c**,**d**) average interaction duration for both saline (blue circles and dots) and testosterone (red circles and dots) treated cleaners as function of client length. Raw data (**a**,**c**) and regression effect plots (**b**,**d**) are presented. Effect plots show partial residuals and prediction lines of each regression model.

**Figure 4:**
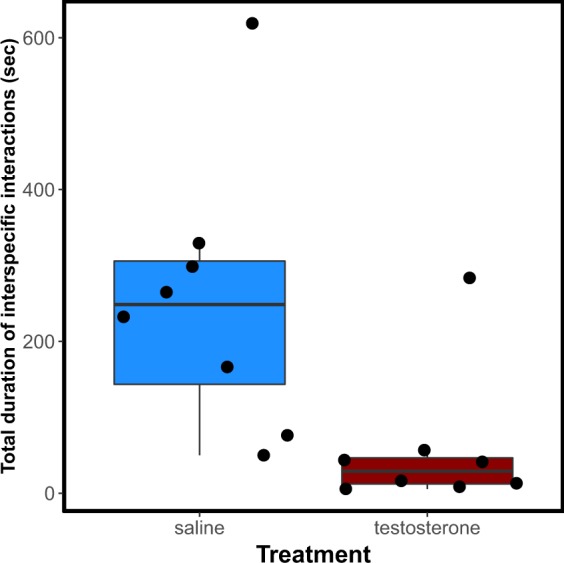
Total time (seconds) spent in interspecific cleaning interactions by cleaner fish treated with saline (blue box) and testosterone (red box).

**Figure 5:**
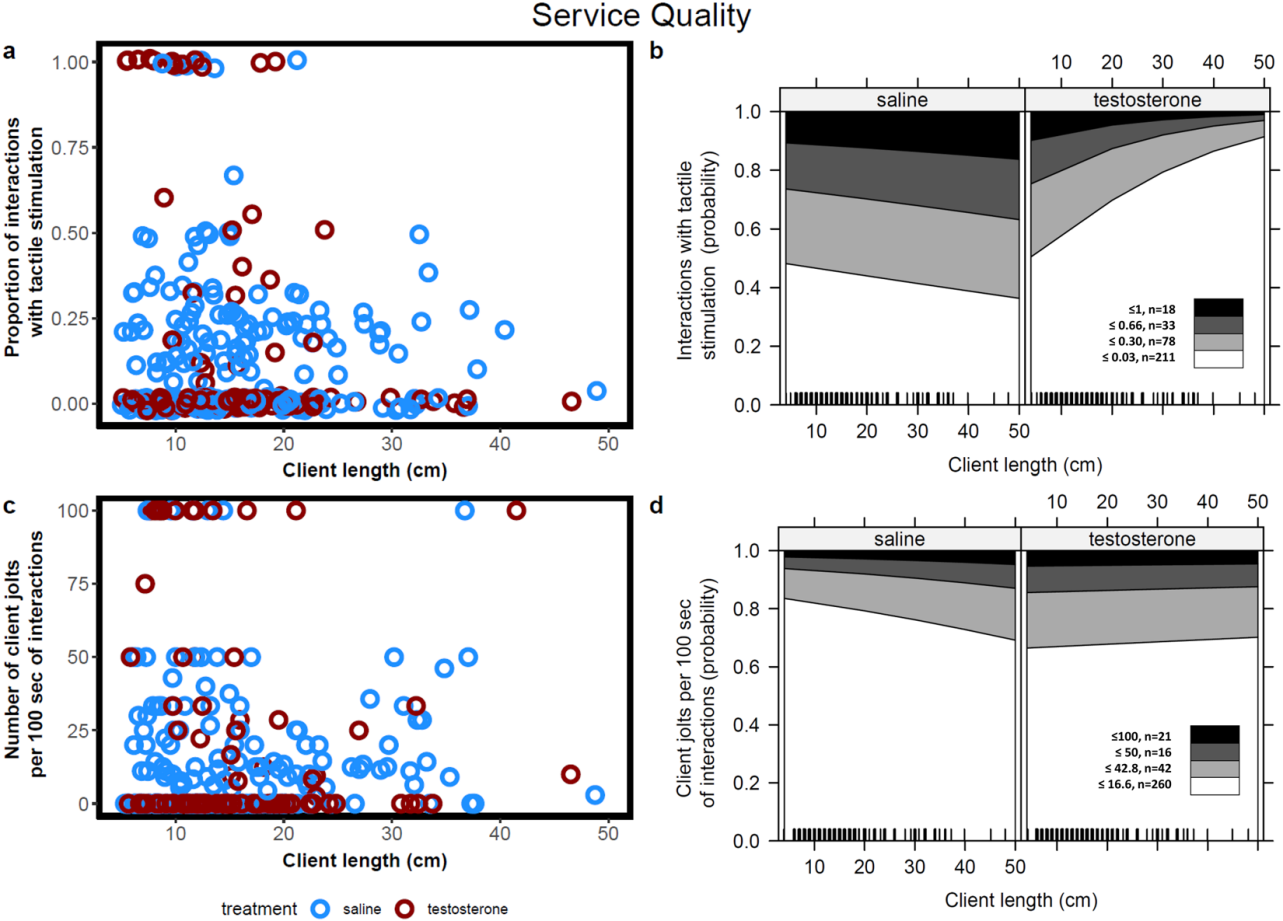
Service quality measured as **(a**,**b**) proportion of interactions with tactile stimulation and (**c**,**d**) number of client jolts per 100 seconds of interactions for saline (blue circles and dots) and testosterone (red circles and dots) treated cleaners as function of client length. Raw data (**a**,**c**) and ordinal regression effect plots (**b**,**d**) are presented. Effect plots show the probabilities across the response categories. The number of data points within a certain category (n) and response categories thresholds are also indicated on the effect plots.

Here we asked two questions: first if testosterone would induce a shift in cleaners’ motivation to engage less in cleaning interactions with clients and second, if it affects the quality of inspection service provided to clients. Concerning motivation, we found that the likelihood of clients being cleaned by testosterone treated cleaners was significantly lower than the likelihood of being cleaned by control cleaners (χ^2^ = 17.7, p = 0.00003, corrected p-value = 0.0002, Fig. 3a,b). Consequently, cleaners treated with testosterone spent less time in cleaning interactions than control cleaners (W = 58, p-value = 0.0074; Fig. 4). Client length did not significantly varied between treatments (F = 0.194, p = 0.6599), but, in both treatments, the likelihood of being cleaned increased with the length of the clients (χ^2^ = 7.96, p = 0.005, corrected p-value = 0.029, interaction term: χ^2^ = 0.36, p = 0.54, corrected p-value = 1, Fig. 3a,b). Average interaction duration was significant shorter for testosterone treated cleaners than for control cleaners (χ^2^ = 34.86, p < 0.0001, corrected p-value < 0.0001, Fig. 3c,d) and, for both treatments, increased with client length (χ^2^ = 31.68, p < 0.0001, corrected p-value < 0.0001, interaction term: χ^2^ = 0.44, p = 0.51, corrected p-value = 1, Fig. 3c,d). Regarding the proportion of tactile stimulation provided, we found no significant differences between treatments (LR = 1.83, p = 0.18), no effect of client length (LR = 0.0003, p = 0.99) and no significant interaction term (LR = 1.77, p = 0.18) (Fig. 5a,b).

**Figure 6:**
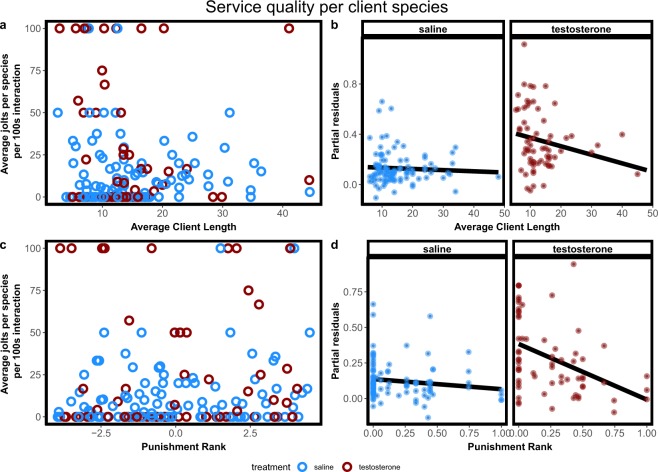
Service quality per client species measured as the average jolts per species per 100 seconds of interactions as function of the average of the client species length (**a**,**b**) and punishment rank (**c**,**d**). Raw data (**a**,**c**) and regression effect plots (**b**,**d**) are presented. Effect plots show partial residuals and prediction lines of each regression model.

For client jolt frequency as a correlate of cheating by cleaners, we considered three hypotheses based on the effects of testosterone on intraspecific social behaviour. First, nothing should change if interactions with clients are modulated by mechanisms that differ from intraspecific interactions. Second, cheating could be generally increased as a result of aggressiveness rise. Third, cheating frequencies may be modulated as a function of the cleaners’ perceived dominance relationships with clients. In the latter case, cleaners could either increase cheating selectively on small client species (linked to physical dominance perception) or selectively on client species that do not punish after cleaner cheating with aggressive chasing (linked to social dominance perception). We found no significant jolt frequency differences between treatments (LR = 1.69, p = 0.19), no effect of individual client length (LR = 0.26, p = 0.61) and no significant interaction term (LR = 0.19, p = 0.67; Fig. 5c,d). When testing if cleaners alter cheating rates selectively for specific client species, we also did not find a significant effect of client species average size on the average proportion of jolts observed (χ^2^ = 1.84, p = 0.17; Fig. 6a,b). However, we found that client species jolt rate was significantly influenced by client species punishment rank (Punishment rank = the average proportion of jolts that generated a punishment reaction by a certain client species; χ^2^ = 9.38, p = 0.002; Fig. 6b,c; see methods for further information) and that there was a significant interaction between treatment and rank (χ^2^ = 6.60, p = 0.01; Fig. 6b,c).

## Discussion

Evidence suggests that testosterone has a key role in social interactions, fairness and bargaining, beyond the typical generalizations of antisocial, and aggressive behaviour; however, these findings have mostly been restricted to human studies^17–24^. Here we show that the administration of testosterone to cleaner fish females resulted in increasing female focus on conspecific activities: a general increase towards the male partner and a selectively aggressive (agonistic) increase towards lower ranking females but also resulted in a decrease of interspecific cleaning activities. Taken together, the results fit the hypothesis that an increase in testosterone mediates a female’s focus on status in a sex-changing species. As also described for humans^19–24^ the effects of testosterone may both enhance cooperation (provision of better cleaning service) and competition in female cleaners, depending on who they interact with.

Importantly, testosterone injections also affected cleaning behaviour. The reduced frequency and duration of interactions by testosterone-treated female cleaners meant that foraging success was significantly reduced. This obvious cost could reflect a basic attention trade-off: females that prioritise intraspecific social interactions may have to repeatedly ignore clients. A second adjustment in testosterone-treated females was the decrease of service quality for client species showing a lower probability to punish cheating by cleaners (when compared to control cleaners, see Fig. 6c,d). Such client species mainly comprise visitors. The provision of good cleaning service to visitor clients yields long-term benefits as it increases the probability of future interactions^47,48^. While frequent cheating of visitors has previously been described as part of a short-term intake maximising strategy (e.g. to bite on large visiting clients instead of behaving as the majority of cleaners that rarely cheat these clients)^46,48–50^, testosterone-treated females fail to show the additional components of that strategy: they also cheat any rarely-punishing resident species and generally fail to increase tactile stimulation to small resident clients as a way to improve their reputation to those passing visitors, that they could exploit. Thus, the changes in the cleaning behaviour by testosterone-treated females do not appear to be adaptive. We propose that cleaners cannot alter their intraspecific social strategies by increasing testosterone levels without altering, and potentially compromising, their interspecific social strategies. In line with our hypothesis, client punishment of cleaners consists of aggressive chasing and is hence very similar to aggressive intraspecific dominance interactions. Therefore, testosterone-treated females may respond more dominantly over non-punishing clients, and increase biting rates, but otherwise become submissive towards punishing clients, thus reducing biting rates.

As reduced foraging efficiency emerges as a cost (even if temporary) of our testosterone treatment, the question arises as to why this effect exists. Indeed, natural selection could lead to a decoupling of mechanisms governing intraspecific social behaviour and interspecific cleaning behaviour so that both types of interactions could, in principle, be optimised independently. This scenario was not supported. Rather, our results fit various previous studies on cleaners, territorial damselfish and surgeonfish suggesting that similar neurobiological pathways that modulate intraspecific social behaviour have been co-opted to influence interspecific social behaviour^51–56^, however with distinct brain and pathways specificities^57,58^. For example, intramuscular injections of the neuropeptide arginine vasotocin made cleaners cease inspecting clients but turning instead their focus to conspecific activities, while those injected with the V1a receptor antagonist were motivated to clean^53^. Similarly, serotonin 1 A receptor antagonist resulted in a decrease of cleaners’ motivation to engage in cleaning but increased female aggressivity towards smaller conspecifics^56^. Moreover, cleaners increased in motivation to clean after exogenous treatment with serotoninergic agonists^56^, a response that is potentially underlined by the increase of diencephalon serotonergic activity^57^. Thus monoamines (serotonin but also dopamine^7,55,56^), neuropeptides^53^ and cortisol^48^ are seemingly implicated in decision-making, with for instance AVT acting as an ontogenetic and punctual switch between cleaning and non-cleaning^59^ and monoamines in contexts of motivation, arousal but all involving mechanisms of neural plasticity and pleotropic modulation of behaviour^60^. In conclusion, our results provide field evidence that testosterone causes pleiotropic effects in social decision-making. This network, which is known to be highly sensitive to sex and stress steroids (and include androgens), neuropeptides and monoamines^8,9,61^, is apparently used for decision-making in both intra- and interspecific social interactions^7,62^.

## Methods

### Field methods

Field manipulations were carried out on 10 different reefs around Lizard Island (Lizard Island Research Station, Australia, 14° 40′S, 145° 28E) between August and September 2011, in which 16 individual female cleaner fish were tested (balanced sampling with cortisol manipulations^48^). Cleaner wrasse’ larval settlement of at these reefs usually happens in November and December^63^ while spawning occurs between October and December^64,65^, which indicates that our field experiments occurred in a “non-spawning” season. As in previous studies^48,53,55,56,66^ manipulations and observations were made by two SCUBA divers, between 10:00 and 16:00 h. Cleaner fish were selected haphazardly across the focus reefs and cleaning stations varied in depth between 1.5 and 12 m. Individuals were captured using a barrier net and measured to the nearest mm (TL-total length). TL of the fish ranged from 6.5 to 8 cm. Body weight was then estimated from a length-weight regression^48,53,55,56^. We then gave the focal female an intra-muscular injection^48,53,55,56,66^ of one of two compounds: a) testosterone dosage 2 µg per gram for body weight (gbw, Sigma – 86500) and b) saline solution (0.9 NaCl). As previous studies^48^, testosterone was first dissolved in 50 µl of ethanol and only then were the solutions made with saline (and left overnight to complete ethanol evaporation). Injection volumes ranged from 20 to 50 µl (gbw). Fish handling was kept under 3 min. Once an individual was released it was then videotaped for the next 45 min, using video cameras in waterproof cases (Sony HDR-XR155)^48,53,55,56,66^. The order of the treatments was randomized for each dive and all treatments used independent cleaner fish. Because this study was done exclusively in field conditions with limitations of time and number of fish used (collecting permit allowance), and also because the removal of blood would equate to animal death (similarly to^48,53,55,56,66^), a putative smaller dosage was chosen based on some previous studies on this subject^67,68^, but mostly on previous studies aiming on other steroids using female cleaner wrasses^48,50,66^. No injury or mortality was detected as a outcome of the injections or behavioural testing.

### Behavioural data collection

As in previous studies^48,53,55,56,66^, video recordings were made from a distance of 2–3 m and during each video analysis, we recorded the following measures: a) client identification (to species) and size (TL) of each visitor (estimated visually to the nearest cm, using the focal cleaner fish’s size estimation as proxy) visiting the cleaning station; b) the number and time spent (in seconds) of a cleaner’s inspection of each client c) the number and duration of tactile stimulation provided (when cleaners touch the body of clients with their body and fins^36^); d) the number of jolt responses by clients (when cleaners bite clients, taking mucus or tissue, these respond with a short body jolt; a behavior that is used as a measure of cheating by cleaner fish^31,69^); and e) conspecific-directed behavior, which included the duration of time spent with conspecific male partner, posing, proving or receiving tactile stimulation, cleaning or simply swimming together (i. parallel swimming^55^), all measures of intraspecific social behavior; and finally antagonistic charges (chases) when one cleaner rapidly advanced toward the other.

### Data analysis

Intraspecific cleaner fish behaviours were grouped in four categories depending on the valence of the behavior (affiliative or agonistic) and towards whom it was directed (males or females). Affiliative behaviors included intraspecific cleaning interactions, posing to a conspecific and swimming with a conspecific. Agonistic behaviors included chasing and attacking a conspecific. We tested for differences between control and testosterone-treated cleaners in the total frequency of conspecific behaviours and total duration of cleaning interactions, as well as in the summed frequency of conspecific affiliative and agonistic behaviours by performing one-sample Wilcoxon tests using the function *wilcox*.*test* in package *stats* (R Core Team, 2017). Interspecific cleaner fish behaviours were analysed in terms of the motivation and service quality of cleaner fish. Concerning motivation, we calculated the likelihood of being cleaned by the client fish as the proportion of total solicitations of cleaning by clients that were successful. We then tested for differences between treatments by performing a Linear Mixed Effect Model (LMM) that included treatment, client length and their interaction a fixed factors, as well as cleaner identity and client species as random factors. We applied an arcsine transformation to the likelihood of being cleaned in order to meet the model assumptions. The model was performed with the function *lmer* from package *lme4*^70^. We also tested for differences between treatments in the average duration of interactions by performing a LMM. The model included treatment, client length and their interaction as fixed factors, as well as cleaner identity and client species as random factors. A logarithmic transformation was applied to the average duration of interactions in order to meet the model assumptions. The analysis was performed with the function *lmer* from package *lme4*^70^. For all the aforementioned models, significance values for fixed factors were obtained with the function *Anova* from package *car*^71^. Concerning cleaner fish service quality, we tested for differences between treatments in the frequency and proportion of time that the cleaner fish spent providing tactile stimulation to clients and in the frequency of jolts per 100 sec of inspection. Since these variables distribution did not meet the assumption of regular mixed models, we tested for differences between treatments by transforming them to ordinal variables and performing Cumulative Link Mixed Models, using the function *clmm* from the package *ordinal*^71^. Significance values for fixed factors were obtained with the function *Anova* from package *car*^71,72^. In all models, cleaner identity and client species were included as random factors. Finally, in order to investigate which factors influenced client species average jolts frequency, we performed a LMM that included treatment, client species average size and client species punishment rank as fixed factors and cleaner identity and client species as random factors. Client species punishment rank was calculated as the average proportion of jolts that generated a punishment reaction by a certain client species. We used the actual and previous data for the calculation of the rank. We performed the model using the function *lmer* from package *lme4*^70^. We applied a logarithmic transformation to the average proportion of jolts in order to meet the model assumptions. Finally, we tested for differences in client length between control and testosterone-treated cleaners by performing a linear model using the function *lm* from package *stats*. Client length was log-transformed to meet the model assumptions. For all models performed, validation was obtained by visual inspection of residuals homogeneity and normality, as well as using the Shapiro-Wilk Normality Test^73^, whenever possible. When only a few assumptions of the models could not be met, results were confirmed with bootstrapping by using the function *confint* from package *stats*^74^. For each group of statistical analyses (intraspecific, motivation and service quality), Holm’s p-values adjustment of multiple comparisons^75^ were performed with the function *p*.*adjust* from package *stats*^74^.

### Ethical note

Animal handling and experimental protocols were first assessed and approved by the Portuguese Veterinary Office (Direcção Geral de Veterinária, license no. 0420/000/000/2009) and then by The University of Queensland Animal Ethics Committee (permit SBS/130/11/FCT).
